# Influencing factors and predictive indicators of return of spontaneous circulation in in-hospital cardiac arrest

**DOI:** 10.3389/fcvm.2025.1514564

**Published:** 2025-04-03

**Authors:** Xiao Wang, Tao Kong

**Affiliations:** ^1^ Department of Cardiology, Fuwai Central China Cardiovascular Hospital, Heart Center of Henan Provincial People’s Hospital, Zhengzhou University, Zhengzhou, China; ^2^ Department of Cardiology, the First Affiliated Hospital of Zhengzhou University, Zhengzhou University, Zhengzhou, China

**Keywords:** in-hospital cardiac arrest, cardiopulmonary resuscitation, return of spontaneous circulation, pan-immune-inflammation value, influencing factors

## Abstract

**Background:**

In-hospital cardiac arrest (IHCA) refers to the occurrence of cardiac arrest in hospitalized patients requiring chest compressions and/or defibrillation, with only about one-third of patients achieving return of spontaneous circulation (ROSC) after cardiopulmonary resuscitation. Pan-immune-inflammation value (PIIV) is an indicator assessing the overall inflammatory status within the body, but the relationship between PIIV and ROSC remains unclear.

**Objective:**

This study aims to analyze the occurrence of ROSC and its influencing factors, and investigate the predictive value of PIIV, in order to provide insights for clinical prevention and treatment.

**Methods:**

Clinical data of IHCA patients admitted to our hospital were retrospectively collected. Patients were divided into the ROSC group and non-ROSC group based on whether spontaneous circulation was restored after cardiopulmonary resuscitation. Multivariate logistic regression was used to analyze factors affecting ROSC, and the receiver operating characteristic (ROC) curve was employed to calculate the area under the curve (AUC) to evaluate the predictive value of PIIV.

**Results:**

168 patients' clinical data were collected, including 62 patients with ROSC and 106 with non-ROSC. The results of multivariate logistic regression analysis showed that the duration of cardiopulmonary resuscitation, adrenaline dosage, blood lactate (Lac), and PIIV were independent influencing factors for ROSC in IHCA patients (*P* < 0.05). The ROC curve analysis revealed that the AUC of PIIV for predicting ROSC in IHCA patients was 0.805 (95% CI: 0.720–0.891), with an optimal cutoff value of 395.3, sensitivity of 83.33%, and specificity of 70.37%.

**Conclusion:**

PIIV demonstrates valuable application in predicting ROSC in IHCA patients.

## 1 Introduction

Cardiac arrest (CA), as a serious medical emergency, refers to the sudden cessation of the heart's pumping action, leading to ineffective blood circulation to various organs throughout the body, especially the brain. Symptoms of cardiac arrest include loss of consciousness, cessation of breathing, and absence of a pulse (1). In the Utstein Resuscitation Registry template, in-hospital cardiac arrest (IHCA) is defined as the provision of chest compressions and/or defibrillation to hospitalized patients experiencing cardiac arrest (2). According to the American Heart Association's guidelines, the incidence of IHCA between 2008 and 2017 has increased to 292,600 cases annually, with at least 9–10 cases occurring per 1,000 hospitalized patients, posing a significant threat to patients' life and health during hospitalization (3). Cardiopulmonary resuscitation (CPR) is one of the primary measures for resuscitating patients experiencing respiratory and cardiac arrest, involving such interventions as endotracheal intubation, chest compressions, defibrillation, aimed at restoring the patient's cardiac circulation autonomously to achieve the goal of resuscitation (4, 5). However, studies indicate that the success rate of cardiopulmonary resuscitation in China is much lower than in countries like the United States (6). Previous research has shown that only about one-third of patients achieve return of spontaneous circulation (ROSC) following cardiopulmonary resuscitation, and some patients may experience poor neurological outcomes leading to resuscitation failure or long-term complications, resulting in adverse prognosis. Therefore, identifying potential indicators that can predict ROSC is critically important for improving patient outcomes (7, 8). While the specific reasons for ROSC remain unclear, the association between inflammation and ROSC has been extensively studied (9, 10). The pan-immune-inflammation value (PIIV), proposed by scholars such as Fuca from the University of Milan in Italy, is an indicator assessing the overall inflammatory status within the body. It combines various inflammatory markers to provide a comprehensive assessment of inflammation, including neutrophil, lymphocyte, monocyte, and platelet counts, directly reflecting the dynamic balance between pro-inflammatory and anti-inflammatory processes (11). Previous studies have demonstrated that PIIV is closely related to the overall mortality and prognosis of patients during hospitalization for acute myocardial infarction (12, 13). However, the relationship between PIIV and ROSC remains unclear. This study aims to analyze the occurrence of ROSC and its influencing factors, investigate the predictive value of PIIV, and provide insights for clinical prevention and treatment.

## 2 Research objects and methods

### 2.1 Research objects

This study is a retrospective investigation. Clinical data of IHCA patients admitted to our hospital from November 2022 to May 2024 were collected. Inclusion criteria comprised patients aged 18 years or older who experienced an IHCA during their hospital stay and subsequently received CPR, with documented initial cardiac rhythms—categorized as either shockable (ventricular fibrillation or pulseless ventricular tachycardia) or non-shockable (asystole or pulseless electrical activity)—thus including both types of cardiac arrest. Additionally, only patients with complete clinical information, including detailed resuscitation data, laboratory parameters, and outcome measures, were enrolled. Exclusion criteria consisted of patients who experienced out-of-hospital cardiac arrest, cases in which resuscitation was not initiated due to family refusal, patients with incomplete or missing key clinical data necessary for analysis, and instances where the initial cardiac rhythm was unclear or undocumented. Patients were categorized into ROSC group and non-ROSC group based on whether spontaneous circulation was restored after cardiopulmonary resuscitation. The criteria for ROSC were as follows: restoration of spontaneous sinus or supraventricular rhythm with a systolic blood pressure ≥50 mmHg (1 mmHg = 0.133 kPa), with the aforementioned criteria sustained for at least 20 min. This study adheres to *Helsinki Declaration* and has been approved by our hospital's medical ethics committee. Informed consent of patients has been obtained for this study.

### 2.2 Data collection

General information, diagnostic and treatment-related data, and resuscitation-related data of the patients were collected, including gender, age, smoking history, alcohol consumption history, past medical history, time of IHCA occurrence, season of occurrence, initial department visited, current department, cause of cardiac arrest, initial monitored rhythm (shockable rhythm, non-shockable rhythm), intubation status, defibrillation, duration of cardiopulmonary resuscitation, amount of epinephrine used. In addition, blood biochemistry parameters were collected at two time points: at admission and after the occurrence of cardiac arrest. These parameters include complete blood count, blood lactate (Lac), albumin, N-terminal pro-B-type natriuretic peptide (NT-proBNP), and blood pH. The admission data served as the baseline inflammatory and biochemical profile, while the post-arrest measurements were also recorded.

### 2.3 Calculation method of PIIV

In-hospital complete blood counts were obtained with an automated hematology analyzer to measure neutrophil count, platelet count, monocyte count, and lymphocyte count. PIIV was calculated as follows: PIIV = (neutrophil count × platelet count × monocyte count)/lymphocyte count (14). In this study, PIIV was measured at two time points: at admission (baseline PIIV) and after the occurrence of cardiac arrest. Unless explicitly noted as “PIIV at admission” (baseline PIIV), all PIIV measurements reported refer to samples obtained post-cardiac arrest.

### 2.4 Statistical analysis

Data analysis in this study was conducted with SPSS 27.0. Continuous data were presented as`*X* ± *S*, and between-group comparisons were performed using independent sample *t*-test. Categorical data were presented as frequencies or percentages, and comparisons were made with chi-square test. Factors influencing non-ROSC were analyzed using multivariate logistic regression analysis. Additionally, the predictive value of PIIV was assessed using the receiver operating characteristic (ROC) curve to calculate the area under the curve (AUC). The significance level was set at *α* = 0.05.

## 3 Results

### 3.1 Case selection

**Figure 1:**
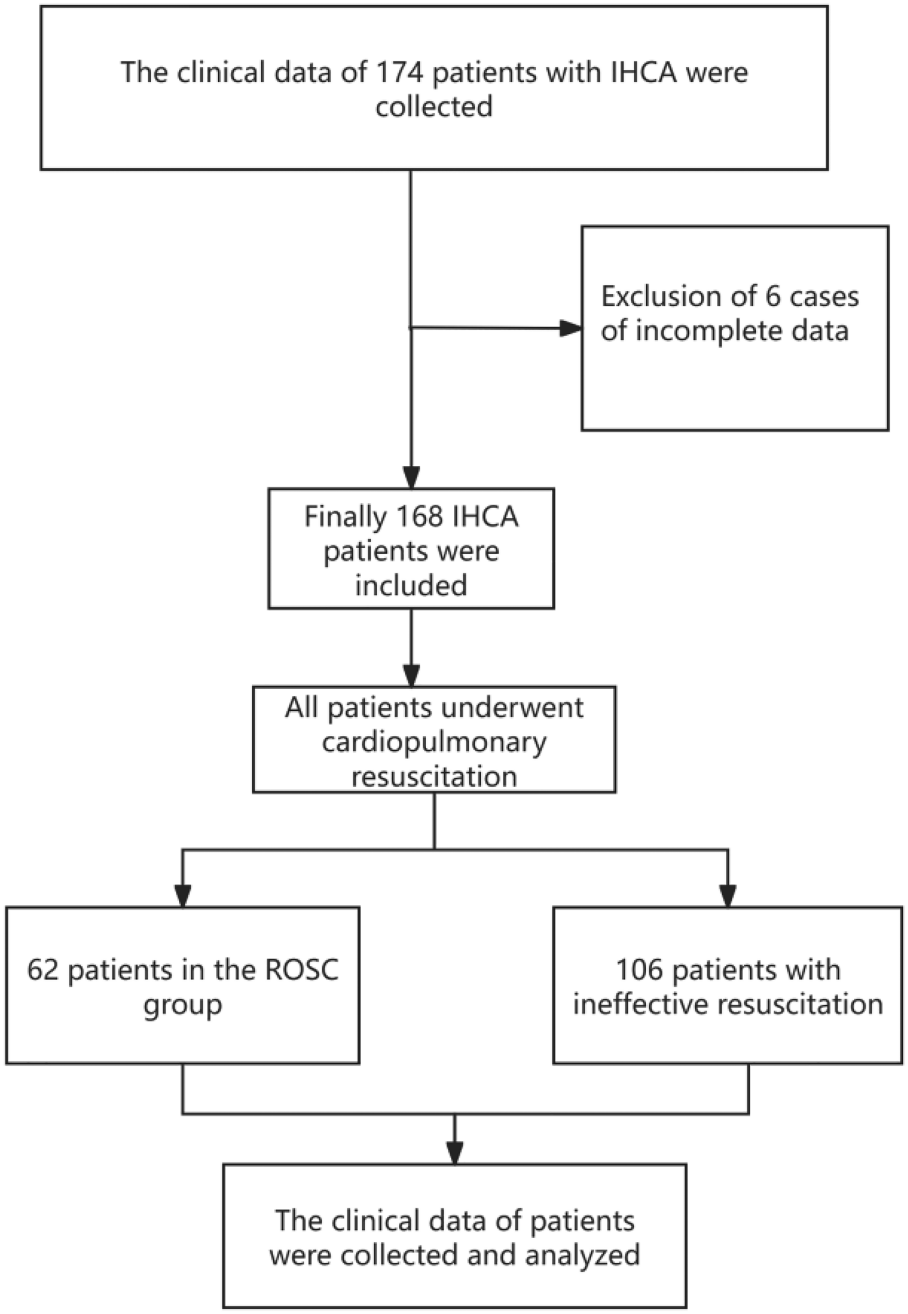
Flowchart of case selection.

174 clinical records of IHCA patients were collected, with 6 cases excluded due to incomplete data. Ultimately, 168 patients were included, comprising 62 patients with ROSC and 106 patients with non-ROSC. The flowchart of case selection is as shown in Figure 1.

### 3.2 General data comparison between non-ROSC group and ROSC group

In the non-ROSC group, there were 65 males and 41 females, with an age range of 59–79 years (mean age: 67.22 ± 9.54 years). In the ROSC group, there were 40 males and 22 females, with an age range of 55–76 years (mean age: 64.19 ± 10.45 years). There were no statistically significant differences in gender, age, smoking history, and other general data between the two groups (*P* > 0.05), as shown in Table 1.

**Table 1:** Comparison of general data between non-ROSC group and ROSC group.

Item		Non-ROSC group (*n* = 106)	ROSC group (*n* = 62)	*χ*^2^/*t* value	*P* value
Gender (cases)	Male	65	40	0.170	0.680
	Female	41	22		
Age (years, $\bar{x}\pm s$)		67.22 ± 9.54	64.19 ± 10.45	1.917	0.057
Smoking history (cases)		31	16	0.230	0.632
Alcohol drinking history (cases)		29	15	0.203	0.653
History of hypertension (cases)		43	24	0.056	0.813
History of coronary heart disease (cases)		14	7	0.132	0.717
History of diabetes (cases)		19	10	0.088	0.766
History of malignant tumors (cases)		18	11	0.016	0.900

ROSC, return of spontaneous circulation.

### 3.3 Comparison of diagnostic and treatment-related data between non-ROSC group and ROSC group

In comparison to the ROSC group, the non-ROSC group had a significantly higher number of instances of initially monitored rhythms that were not shockable (*P* < 0.05). There were no statistically significant differences in other diagnostic and treatment-related data such as IHCA occurrence time and season between the two groups (*P* > 0.05), as shown in Table 2.

**Table 2:** Comparison of diagnostic and treatment-related data between non-ROSC group and ROSC group (cases).

Item		Non-ROSC group (*n* = 106)	ROSC group (*n* = 62)	*χ*^2^ value	*P* value
Time of IHCA occurrence	8:00 a.m.–7:59 p.m.	41	33	3.359	0.067
	8:00 p.m.–7:59 a.m.	65	29		
Season of IHCA occurrence	Spring/Summer	55	33	0.028	0.867
	Autumn/Winter	51	29		
Initial symptoms of CA	With prodromal symptoms	44	23	0.318	0.573
	Without prodromal symptoms	62	39		
Location of IHCA	Internal medicine	31	13	2.256	0.324
	Surgery	16	14		
	Emergency/ICU/Other	59	35		
Cause of CA	Cardiac origin	54	29	0.272	0.602
	Non-cardiac origin	52	33		
Initial rhythm monitoring	Shockable rhythm	10	13	4.404	0.036
	Non-shockable rhythm	96	49		

ROSC, return of spontaneous circulation; IHCA, in-hospital cardiac arrest; CA, cardiac arrest.

### 3.4 Comparison of resuscitation-related data between non-ROSC group and ROSC group

In comparison to the ROSC group, the non-ROSC group had significantly more cases with defibrillation, CPR duration >30 min, administration of epinephrine, and epinephrine usage >5 mg, with statistically significant differences (*P* < 0.05). There were no statistically significant differences in the number of cases with endotracheal intubation between the two groups (*P* > 0.05), as shown in Table 3.

**Table 3:** Comparison of resuscitation-related data between non-ROSC group and ROSC group (cases).

Item		Non-ROSC group (*n* = 106)	ROSC group (*n* = 62)	*χ*^2^ value	*P* value
Endotracheal intubation	Yes	32	28	5.133	0.077
	No	46	17		
	Already with an open airway measure	28	17		
Defibrillation	Yes	19	21	5.484	0.019
	No	87	41		
CPR duration	≤30 min	33	42	21.221	<0.001
	>30 min	73	20		
Epinephrine administration	Yes	99	52	3.902	0.048
	No	7	10		
Epinephrine dose	≤5 mg	58	48	8.659	0.003
	>5 mg	48	14		

ROSC, return of spontaneous circulation.

### 3.5 Comparison of blood biochemical parameters between non-ROSC group and ROSC group

Compared to the ROSC group, the non-ROSC group exhibited significantly higher levels of Lac, NT-ProBNP, and PIIV, with statistically significant differences (*P* < 0.05). There were no statistically significant differences in such parameters as albumin, blood glucose, and PIIV at admission between the two groups (*P* > 0.05), as shown in Table 4.

**Table 4:** Comparison of blood biochemical parameters between non-ROSC group and ROSC group $\left(\bar{x}\pm s\right)$.

Item	Non-ROSC group (*n* = 106)	ROSC group (*n* = 62)	*t* value	*P* value
Lac (mmol/L)	7.28 ± 2.25	5.54 ± 1.94	5.082	<0.001
Albumin (g/L)	34.96 ± 2.35	35.47 ± 2.89	1.245	0.215
NT-ProBNP (μg/L)	2.84 ± 0.65	0.97 ± 0.39	20.575	<0.001
Blood pH	7.39 ± 0.89	7.22 ± 0.95	1.165	0.246
Blood glucose (mmol/L)	10.27 ± 1.69	10.08 ± 1.74	0.696	0.488
PIIV at admission	218.56 ± 39.65	226.86 ± 38.91	1.318	0.189
PIIV	466.38 ± 62.17	318.65 ± 49.54	15.972	<0.001

ROSC, return of spontaneous circulation; Lac, lactate; NT-ProBNP, N-terminal pro-B-type natriuretic peptide; PIIV, pan-immune-inflammation value.

### 3.6 Multivariate logistic regression analysis of factors influencing ROSC in IHCA patients

Multivariate logistic regression analysis was performed with variables showing statistically significant differences (*P* < 0.05) in the univariate analysis as independent variables and the occurrence of ROSC in IHCA patients as the dependent variable. The results indicate that the duration of CPR, epinephrine dosage, lactate levels (Lac), and PIIV are independent influencing factors for ROSC in IHCA patients (*P* < 0.05), as shown in Table 5.

**Table 5:** Multivariate logistic regression analysis of factors influencing ROSC in IHCA patients.

Factor	*β*	SE	Ward *χ*^2^	*P*	OR	95% CI
Non-shockable rhythm	−0.582	0.452	1.656	0.499	0.559	0.230–1.356
Defibrillation	0.212	0.314	0.455	0.165	1.236	0.668–2.287
CPR duration ≤30 min	−1.214	0.408	8.854	0.008	0.297	0.133–0.661
Epinephrine administration	−0.460	0.336	1.878	0.441	0.631	0.327–1.219
Epinephrine dose ≤5 mg	−1.317	0.354	13.836	<0.001	0.268	0.134–0.536
Lac	−0.944	0.322	8.598	0.007	0.389	0.207–0.731
NT-ProBNP	−0.243	0.175	1.934	0.395	0.784	0.556–1.105
PIIV	−0.402	0.108	13.853	<0.001	0.669	0.541–0.827

### 3.7 ROC curve analysis results of PIIV for predicting ROSC in IHCA patients

**Figure 2:**
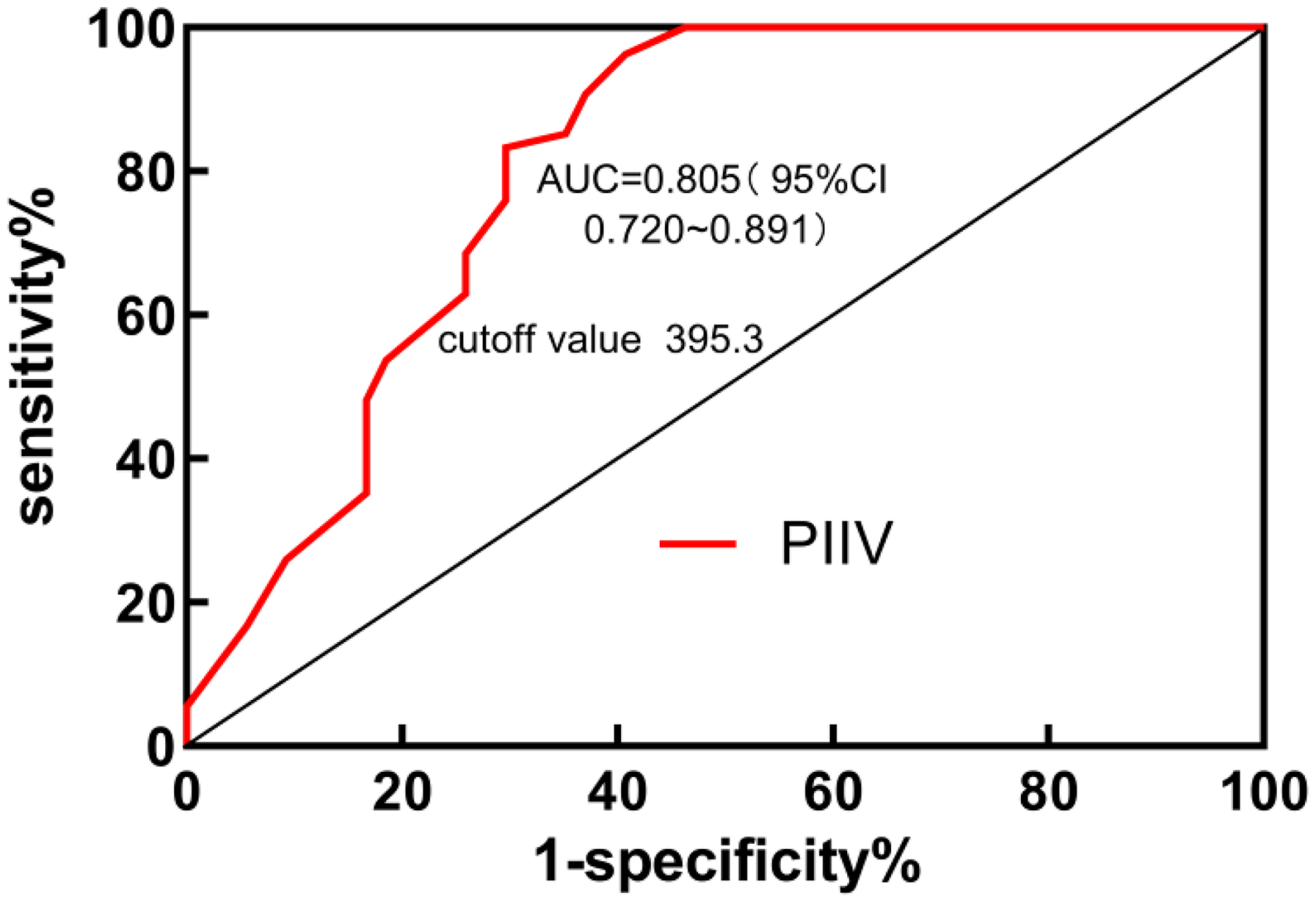
ROC curve of PIIV for predicting ROSC in IHCA patients.

The ROC curve analysis results indicated that the Area Under the Curve (AUC) for PIIV in predicting ROSC in IHCA patients was 0.805 (95% CI: 0.720–0.891). The optimal cutoff value was 395.3, with a sensitivity of 83.33% and specificity of 70.37%, as shown in Figure 2.

## 4 Discussion

In this study, a total of 168 IHCA patients who underwent CPR were included, with 62 cases achieving ROSC, accounting for 36.90%. This proportion is slightly higher than the data reported in the Swedish CPR registry at 35.6% (15). This difference may be attributed to the relatively better overall health status of the patients included in this study. Additionally, the study period for patient inclusion in this research ranged from November 2022 to May 2024, whereas the Swedish study was conducted in 2020. CPR outcomes typically evolve over time with advancements in technology and improvements in treatment methods, such as enhanced CPR techniques, drug utilization, and equipment, which may contribute to an increased ROSC rate. Numerous studies have demonstrated that by predicting the likelihood of ROSC early on, medical teams can better adjust treatment strategies and allocate resources, thereby enhancing patients' chances of recovery and overall prognosis. Simultaneously, by predicting the likelihood of ROSC, healthcare personnel can exercise caution when intervening with low-prognosis patients, avoiding excessive treatments for patients with unclear benefits. This approach helps reduce unnecessary medical interventions and their associated burdens (16, 17).

In this study, the duration of CPR, epinephrine dosage, and Lac were identified as independent influencing factors for ROSC in IHCA patients, aligning closely with findings from previous research (18). The optimal duration of CPR remains a topic of debate in the international arena. High-quality uninterrupted chest compressions significantly impact the success rate of ROSC following CPR. However, if CPR is prolonged beyond the body's tolerance, it can lead to hypoxia, ischemia-reperfusion injury in vital organs such as the heart, brain, and lungs, resulting in irreversible damage that affects the success rate of ROSC (19). A study by Coppler indicated that the type of brain injury is associated with the duration of CPR, where the duration serves as a surrogate marker for the severity of hypoxic-ischemic brain injury. Longer CPR durations correlate with lower survival rates (20). Research by Okubo and colleagues, based on extensive multicenter in-hospital cardiac arrest registry data, highlighted a gradual decline in survival rates and favorable functional outcomes with prolonged CPR durations. Beyond 39 min of CPR, survival rates dropped to below 1%, and at 32 min of CPR, the probability of favorable functional outcomes also fell below 1%. This suggests that while CPR can be effective within a certain timeframe, the likelihood of successful recovery significantly diminishes after a specific duration (21). The optimal duration of CPR for successful outcomes remains unclear, underscoring the necessity for large-scale research to comprehend the impact of CPR duration on survival rates.

Numerous studies have indicated that the use of epinephrine can increase the rate of ROSC, with epinephrine being recommended as the preferred rescue medication for CPR by many guidelines (22, 23). However, continuous use of more than 5 mg of epinephrine may increase cardiac oxygen consumption. While it can elevate blood pressure and perfusion in the early stages of resuscitation, excessive use under hypoxic conditions may lead to damage to myocardial cells, especially in cases of cardiac hypoxia, resulting in post-resuscitation cardiac dysfunction (24). Some studies suggest that excessive use of epinephrine may be associated with poorer functional outcomes. While it may increase the occurrence of ROSC, it could potentially have adverse effects on survival quality and functional outcomes post-recovery (25). CPR guidelines typically recommend a dose of 1 mg (1:10,000 dilution) of epinephrine per administration, with repeat doses every 3–5 min if circulation has not been restored. The total dose used should be within an appropriate range. This highlights the need in clinical practice to balance the potential benefits and risks of using epinephrine, adjusting medication strategies based on the patient's specific condition (26). Lactic acid is a metabolic byproduct of hypoxia and poor tissue perfusion. During cardiac arrest, the cessation of cardiac pumping leads to severe systemic tissue hypoxia, particularly affecting oxygen supply to vital organs such as the brain and heart, resulting in elevated lactate levels. High lactate levels reflect the extent of tissue hypoxia and metabolic disruption, thus correlating with the severity of cardiac arrest and the difficulty of resuscitation (27). Research by Li et al. highlighted that high lactate levels are an independent risk factor for mortality in patients undergoing CPR. Elevated lactate levels typically indicate severe tissue hypoxia and difficulty in circulatory restoration, thereby reducing the likelihood of ROSC (28).

Cardiac arrest and the resuscitation process can trigger significant systemic inflammatory responses. This inflammation includes elevated markers such as cytokines, interleukins, CRP, and other indicators. Previous studies have indicated that post-cardiac arrest, tissue hypoxia, and reperfusion injury can lead to local and systemic inflammatory responses (29). A high pan-immune inflammation index signifies elevated levels of inflammation in the body, which may exacerbate tissue damage, impact the effectiveness of cardiac recovery and rehabilitation, and reduce the success rate of ROSC. Neutrophils are the most abundant white blood cells in the circulatory system and can reflect the body's systemic or local inflammatory state. They regulate the inflammatory microenvironment by releasing cytokines, chemokines, and growth factors, thereby promoting the body's inflammatory response. Platelets are closely related to various inflammatory processes as they secrete and express many pro-inflammatory and anti-inflammatory cell molecules after activation through Toll-like receptors binding with pathogens, playing a role in antigen presentation. Lymphocytes play various roles in the inflammatory response through mechanisms such as regulating immune responses, directly killing infected cells, producing antibodies, and secreting cytokines. Their role is crucial for effectively clearing pathogens and maintaining immune balance, but improper or excessive inflammatory responses may also lead to tissue damage and disease progression (30).

The PIIV integrates these indicators and can comprehensively reflect the body's inflammatory status. A high inflammatory state is typically associated with poorer ROSC outcomes because inflammation can cause further damage to the heart and other vital organs. Research by Liu et al. indicated that compared to the systemic immune inflammation index (SII), PIIV has good predictive value for the prognosis of acute myocardial infarction patients undergoing coronary artery revascularization (31). A meta-analysis study demonstrated that PIIV can predict overall survival and progression-free survival in breast cancer patients (32). The results of this study confirm the value of PIIV in predicting ROSC in IHCA patients, suggesting that PIIV is a more reliable predictor for ROSC. PIIV as an independent influencing factor for ROSC in IHCA patients is because it comprehensively reflects the systemic inflammatory response post-cardiac arrest, which significantly impacts cardiac resuscitation and patient prognosis. A high level of inflammation usually indicates poorer tissue recovery and prognosis, thus serving as an effective predictor for ROSC. In addition to PIIV, other inflammatory markers like the systemic immune-inflammation index (SII) have been investigated in various clinical contexts. SII, which is calculated as (platelet count × neutrophil count) divided by lymphocyte count, has been widely recognized as a predictor of outcomes in several patient populations. Compared to SII, PIIV incorporates monocyte count into its calculation, potentially offering a more comprehensive assessment of the systemic inflammatory response. Our findings suggest that PIIV is a robust predictor of ROSC in IHCA patients, and its prognostic performance may be enhanced by this additional parameter. However, direct comparisons between PIIV and SII in the context of IHCA are still limited. Future studies should aim to directly compare these indices in order to clarify their respective roles and to determine whether the inclusion of monocyte count in PIIV confers any significant advantage over SII in predicting clinical outcomes following cardiac arrest (33, 34).

One important limitation of our study is the variability in clinical protocols across different hospital departments. In our retrospective analysis, resuscitation procedures, medication usage, and post-resuscitation care were not standardized across all departments. For instance, some departments may have adopted more aggressive resuscitation strategies or had greater access to advanced life support resources, while others followed more conservative protocols due to resource constraints or differences in staff training. This heterogeneity could have influenced the observed outcomes, including ROSC rates, and may confound the relationship between inflammatory markers such as PIIV and ROSC. Additionally, our study included only those IHCA patients who underwent resuscitation attempts. As a result, the findings are applicable solely to the subset of patients deemed eligible for and who received resuscitation, and this selection bias may affect the overall characteristics and outcomes reported. Future studies should aim to standardize clinical protocols across departments or incorporate departmental variables into multivariate regression models, as well as include a broader patient population, to reduce these biases and improve the generalizability of the results. Furthermore, the retrospective design of our study inherently limits causal inference and may introduce additional selection biases. We also acknowledge that key variables—such as patient comorbidities, variability in resuscitation quality, and post-resuscitation care—were not comprehensively analyzed, which could influence the observed outcomes. Moreover, as resuscitation measures and medications continue to evolve, it is imperative to include additional factors influencing ROSC in future multivariate regression analyses.

In conclusion, PIIV has significant practical value for predicting ROSC in IHCA patients. It not only reflects the inflammatory and immune challenges faced by patients during resuscitation but also provides clinicians with a quantifiable tool to better manage resuscitation strategies, assess prognosis, and optimize patient care. By considering PIIV comprehensively, physicians can make more effective clinical decisions, enhancing the survival and recovery quality of in-hospital cardiac arrest patients.

## Data Availability

The original contributions presented in the study are included in the article/Supplementary Material, further inquiries can be directed to the corresponding author.
